# Involvement of Mast Cells in the Pathophysiology of Pain

**DOI:** 10.3389/fncel.2021.665066

**Published:** 2021-06-10

**Authors:** Lijia Mai, Qing Liu, Fang Huang, Hongwen He, Wenguo Fan

**Affiliations:** ^1^Department of Anesthesiology, Guanghua School of Stomatology, Hospital of Stomatology, Sun Yat-sen University, Guangzhou, China; ^2^Guangdong Provincial Key Laboratory of Stomatology, Institute of Stomatological Research, Sun Yat-sen University, Guangzhou, China

**Keywords:** mast cells, pain, hyperalgesia, inflammation, immunoregulation

## Abstract

Mast cells (MCs) are immune cells and are widely distributed throughout the body. MCs are not only classically viewed as effector cells of some allergic diseases but also participate in host defense, innate and acquired immunity, homeostatic responses, and immunoregulation. Mounting evidence indicates that activation of MCs releasing numerous vasoactive and inflammatory mediators has effects on the nervous system and has been involved in different pain conditions. Here, we review the latest advances made about the implication of MCs in pain. Possible cellular and molecular mechanisms regarding the crosstalk between MC and the nervous system in the initiation and maintenance of pain are also discussed.

## Introduction

Pain is a hallmark of inflammation that can be either protective or detrimental during acute or chronic stages. The development and maintenance of chronic pain are involved in neuronal sensitization (Ji et al., 2016). It has long been postulated that interactions between the nervous system and immune system contribute to the pathophysiology of pain. Following intense noxious stimulation, neuropeptides and neurotransmitters released by nociceptors result in neurogenic inflammation and the recruitment of immune cells, whereas infiltrated immune cells release mediators to enhanced responsiveness of sensory neurons. Such positive feedback loops may underlie pain induction (Liu et al., 2021).

Considerable evidence suggests that mast cells (MCs), effectors of innate immunity and local inflammation, regulate pain signaling, for example, by secreting mediators that activate nearby nerves based on their histological proximity (Chompunud Na Ayudhya et al., 2020; Aguilera-Lizarraga et al., 2021). Here, we discuss the role of MCs in pain initiation and maintenance *via* MC-neuron crosstalk. Possible molecular mechanisms and resolution of pain associated with MC are demonstrated. Importantly, the identification of the pathological role of MCs in neuroimmune interactions will provide us novel strategies operative in pain.

## Mast Cell Basics

**Figure 1:**
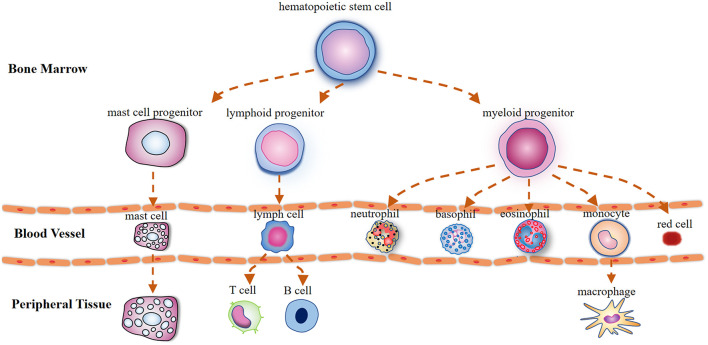
Illustration outlining the mast cells (MCs) differentiation trajectories. Mast cells, lymphocytes, and myeloid cells are derived from pluripotent hematopoietic progenitors in the bone marrow. Unlike basophils that attain their maturity in the circulation, mast cell precursors circulate in the bloodstream as immature cells and are transited out to the peripheral tissues where they mature under the influence of growth factors.

MCs derive from CD34/CD117-expressing multipotent hematopoietic precursor cells in the bone marrow, which circulate in the bloodstream and are transited out of the circulation to the peripheral tissues where they attain their maturity (Metcalfe et al., 1997; Figure 1). Mature MCs can exert instant effects on vascular function (Albert-Bayo et al., 2019) and sensory neurons as they are in close proximity to vasculature and nerve fibers innervating derma (Morellini et al., 2018), visceral organs (Barbara et al., 2004), meninges (Levy et al., 2007; Hassler et al., 2019), brain parenchyma (Ocak et al., 2019), and hypothalamus (Edvinsson et al., 1977).

MCs can be activated through a variety of mechanisms. Of these, allergens and pathogens acting on their respective receptors expressed on MCs, such as the high-affinity immunoglobulin E receptor and toll-like receptor, represents the classical model of MC activation (González-de-Olano and Álvarez-Twose, 2018). Notably, MCs can be activated by membrane receptors that can not only detect thermal and physical stimuli [e.g., the transient receptor potential vanilloid (TRPV) family] (Zhang D. et al., 2012; Solís-López et al., 2017), but also detect a variety of endogenous mediators, including neuropeptides and neurotransmitters released by nociceptive neurons [e.g., Mrgprb2/X2, a G protein-coupled receptor responsive to substance P (SP)] (Green et al., 2019).

Following activation, MCs release their granule-stored mediators and then secret re-synthesized granules as a late response, called “*de novo* synthesis” (Vukman et al., 2017). The former process is termed “degranulation”, in which MCs release pre-formed granules within minutes. These mediators include biogenic amines (e.g., histamine and serotonin), proteases (e.g., tryptase and chymase), proteoglycans (e.g., heparin) tumor necrosis factor alpha (TNFα), leukotrienes, cytokines, and chemokines that facilitate the migration of other immune cells (González-de-Olano and Álvarez-Twose, 2018). They can be recognized in tissues with toluidine blue staining due to the large cytoplasmic granules (mainly heparin) in cells (Eady, 1976).

## Mast Cell Involved in Painful Conditions

MCs are located in the vicinity of nociceptive C-fibers and may interact with nerve endings through the “synapse like” connection (Suzuki et al., 2004). Increased MCs were observed in patients with headaches (Friesen et al., 2018), non-cardiac chest pain (Lee et al., 2014), and self-injurious behavior-associated pain (Symons et al., 2009). Pain-like behaviors have been found to be MC-associated, including models of post-fracture nociception (Li et al., 2012), cancer pain (Lam and Schmidt, 2010; Yu et al., 2019), postoperative pain (Oliveira et al., 2013), fibromyalgia (muscle pain; Theoharides et al., 2019), sickle cell anemia-associated pain (Vang et al., 2015) and visceral hypersensitivity, as is indicated in irritable bowel syndromes (Di Nardo et al., 2014), chronic pelvic pain (Done et al., 2012), interstitial cystitis (IC; Wang et al., 2016; Martin Jensen et al., 2018), and neonatal maternal separation (Chen et al., 2021). Mastocytosis, characterized by constitutive hyperactivity of MC, is often accompanied by pain syndromes (Giannetti and Filice, 2021). Additionally, MC stabilizers significantly attenuate hyperalgesia in inflammatory pain models induced by formalin (Nakajima et al., 2009), potamotrygon venom (Kimura et al., 2018), nerve growth factor (NGF), and dynorphin (Kissel et al., 2017).

Taken as a whole, the results indicate that MCs play an important role in different painful conditions, although some studies showed that depletion or stabilization of MC did not display pain-relieving effect in models induced by complete Freund’s adjuvant, carrageenan, formalin, NGF, or nociceptin/orphanin (McDougall and Larson, 2006; Xanthos et al., 2011; Lopes et al., 2017; Magnusdottir et al., 2018). Whether MC plays a critical role in nociceptive processing remains to be elucidated.

## Mechanistic Insights into The Dialog Between Neuron and Mast Cell

**Figure 2:**
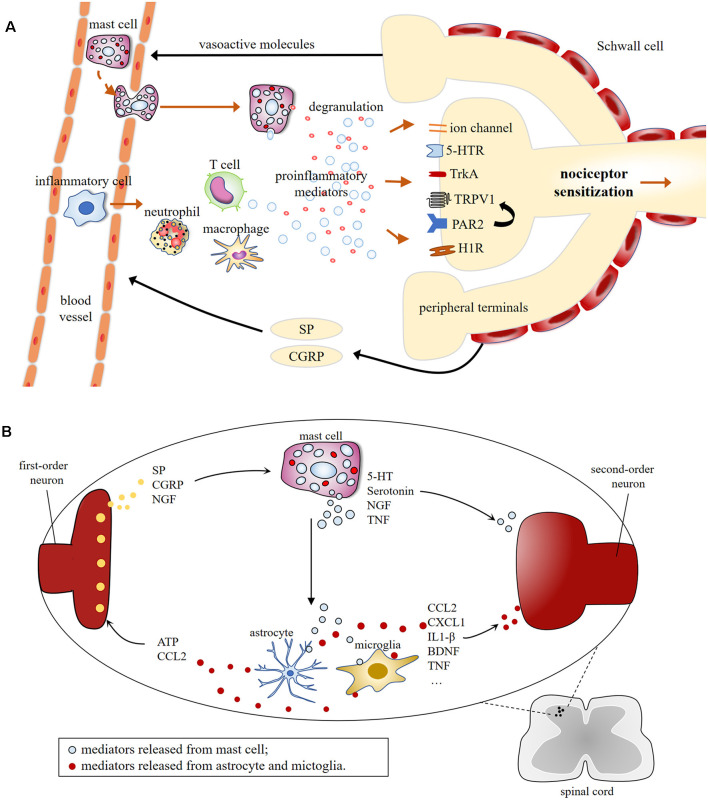
**(A)** Schematic illustration of mast cell involvement in peripheral sensitization in the terminals of nociceptive primary afferents. Mast cell degranulation induces the production of proinflammatory mediators [e.g., 5-HT, TNF, nerve growth factor (NGF), histamine, tryptase], resulting in nociceptive neurons release vasoactive neuropeptides, which in turn, leads to the recruitment of immune cells, including mast cells, macrophage, neutrophil, T cell, etc. This leads to the possibility of positive feedback loop, which could lead to chronic pain. **(B)** Molecular mechanisms of central sensitization induced by mast cells degranulation in first-order excitatory synapses, where communications between neuronal and non-neuronal cells occur. Central terminals of nociceptors release inflammatory factors that activate the second-order neurons and non-neuronal cells including mast cells, which induces neuronal activation *via* producing proinflammatory cytokines and chemokines [e.g., TNF, interleukins (IL)-1β, CCL2, CXCL1], and granular components, such as 5-HT and serotonin.

MCs are well recognized for their sufficient role in inflammation but much less is known about their contributions to pain pathways. MC may increase the excitability of nociceptors by releasing pro-nociceptive molecules, whose receptors are expressed on sensory neurons (Loewendorf et al., 2016). Mediators released by nociceptive sensory neurons, in turn, regulate the maturation, recruitment, and degranulation of MCs through the activation of their respective membrane receptors on MCs (Serhan and Basso, 2019; Koyuncu Irmak et al., 2019; Figure 2).

### Neuropeptides

Neuropeptides, critical inducers of neurogenic inflammation, are primarily released from nociceptors following intense noxious stimulation and/or activation of different molecular sensors, such as TRP channels (e.g., TRPV1; Sousa-Valente and Brain, 2018; Yakubova and Davidyuk, 2021), G protein-coupled receptors [proteinase-activated receptor 2 (PAR2) and Mrgprb2] (Wei et al., 2016), sodium channels (Nav1.9; Bonnet et al., 2019), and mechanosensitive Piezo receptors (Mikhailov et al., 2019).

SP and calcitonin gene-related peptide (CGRP) are two of the pivotal neuropeptides implicated in neurogenic inflammation and pain. Recent evidence suggests that SP promotes the recruitment of innate immune cells and the release of pro-inflammatory mediators *via* activation of the Mrgprb2 receptor expressed by MCs (Green et al., 2019). A recent report reveals a regulatory effect of CGRP in MCs using RNA-sequencing, in which differentially expressed genes are enriched in biological processes associated with transcription, MC activation, and proliferation after CGRP treatment (Sun et al., 2020). Although MCs abundantly express receptors for neuropeptides (Le et al., 2016), however, many neuropeptides have less well-defined roles in MC-mediated pain.

In turn, MCs may exacerbate inflammation and pain signals *via* modulating SP production. MCs reside in the microenvironment where SP- immunoreactive nerve fibers are located and modify the degradation of SP by releasing tryptase and chymase (Caughey et al., 1988). Pharmacological inhibition on MCs significantly reduces the release of SP and ameliorates hyperalgesia in sickle mice (Vincent et al., 2013). Identification of the modulatory effects of MCs on SP and CGRP may provide insights into the neuro-immune interaction, but not exclusively, pain hypersensitivity.

### Serotonin

Serotonin, or 5-hydroxytryptamine (5-HT), is a neurotransmitter that distributes mainly in the central nervous system and it is involved in the regulation of numerous behavioral and physiological processes, such as perception, memory, and mood (Bamalan and Al Khalili, 2020). Recent studies suggest that serotonin can be released from peripheral MC and promote pain during tissue injury (Sommer, 2004).

The expression level of 5-HT was upregulated in pain models induced by acute inflammation (Nakajima et al., 2009), surgery (Oliveira et al., 2011), and migraine (Koroleva et al., 2019), which can be significantly attenuated by MC stabilizer or MC deficiency. Patients with abdominal pain showed a significantly increased release of 5-HT, which has a significant correlation with MCs counts and the severity of pain (Taylor et al., 2010; Cremon et al., 2011).

5-HT is also a powerful neuromodulator with a receptor-dependent effect. Several subtypes of serotonin receptors, such as 5-HT(1)A (Coelho et al., 1998), 5-HT(3) (Yan et al., 2014), and 5-HT(2A) receptors (Oliveira et al., 2011), have been found associated with nociceptive responses mediated by MC. Selected tricyclic antidepressants, capable of inhibiting 5-HT secretion from MCs, are well introduced in chronic pain treatment, which expand our understanding of mechanisms underlying the pathophysiology of pain (Ferjan and Lipnik-Stangelj, 2013).

### Histamine

Histamine is present within all bodily tissues, stored in secretory vesicles that are released by MCs and basophils. Histamine regulates various physiological and pathological processes, such as autoimmune conditions, vasodilation, hematopoiesis, and neurotransmission (Obara et al., 2020), which are facilitated by binding to histamine H_ 1_, H_2_, H_3_, and H_4_ receptors that differ in their tissue expression patterns and functions (Obara et al., 2020; Patel and Mohiuddin, 2020).

Accumulating evidence indicates that MC-derived histamine serves as mediator to pain. Treatment with MC stabilizers and/or histamine antagonists significantly ameliorates vincristine/paclitaxel-induced hyperalgesia (Gao et al., 2016; Schneider, 2017). Blockade of H1 receptor in pain models with increased MCs infiltration inhibits or reduces prostatitis-associated pelvic pain (Done et al., 2012), visceral hypersensitivity (Barbara et al., 2007), venom-induced mechanical allodynia (Lauria et al., 2018), and post-operative nociception (Oliveira et al., 2011). H_2_ receptors also indicated in hyperalgesia and allodynia mediated by MC histamine in inflammatory pain (Massaad et al., 2004), vincristine-induced neuropathic pain (Jaggi et al., 2017), and IC pain (Rudick et al., 2008). Given the efficacy of histamine antagonists in treating hyperalgesia, inhibition on MC degranulation may provide a promising target in pain control (Obara et al., 2020).

### Tryptase

Tryptase is a trypsin-like serine protease produced by MCs. It serves as a marker of MC activation. The release of tryptase has been proven to be attributed to activation of Kit receptor in MCs (Grimbaldeston et al., 2005; Ammendola et al., 2013; Chen et al., 2021).

MC tryptases are essential for inflammation and nociceptive responses (Hoffmeister et al., 2011; Borbély et al., 2016). Clinically, there was a significant correlation between the intensity of pain and tryptase levels in patients who are with the complex regional pain syndrome (Huygen et al., 2004). Increased level of tryptase in the incised tissue was detected in most patients who were undergoing moderate-to-severe pain for up to 1 month (Pepper et al., 2013). Tryptase may be involved in pain through cleaving and activating its receptor PAR2 expressed on sensory neurons (Anaf et al., 2006; Bunnett, 2006). As pretreatment with PAR2 antagonist was capable of attenuating chronic visceral hyperalgesia (Roman et al., 2014), preventing postoperative nociception (Oliveira et al., 2013), and abolishing cancer-dependent allodynia (Lam and Schmidt, 2010).

Some studies revealed that tryptase-PAR2 may affect neurogenic inflammation and pain transmission *via* regulating the activity of TRP ankyrin 1 and TRPV1, TRPV4 channels of sensory neurons (Dai et al., 2004, 2007; Zhao et al., 2014), by phospholipase C, protein kinase A, and protein kinase C-dependent mechanisms (Chen et al., 2011). Moreover, MC tryptase activates neutrophil (de Almeida et al., 2020) and microglia (Zhang S. et al., 2012), which are important culprits for inflammation and exerts an active role in pain (Tsuda, 2018). MC tryptase has been implicated in peripheral and central sensitization, albeit there remain large gaps in our knowledge about the tryptase-mediated mechanism of nociception.

### Cytokines

Cytokines are synthesized mainly by the immune and nervous system and are responsible for the regulation of differentiation, inflammation, immune responses, cell apoptosis, and necrosis *via* transmitting signals between cells (Totsch and Sorge, 2017; Zahari et al., 2017). Additionally, cytokines contribute significantly to pain arising from nociceptor activation. A range of cytokines, including TNFα, interleukins (IL)-1beta, IL-6, IL-17, granulocyte macrophage colony-stimulating factor (GM-CSF), have been shown to play prominent roles in sensitizing neuronal cells *via* their specific receptors (Cook et al., 2018).

Non-neuronal cells, such as MCs, monocytes, lymphocytes, are producers of TNF (Grivennikov et al., 2005). A previous finding has identified MCs as an important source of both preformed and immunologically inducible TNF implicated in different biological responses (Gordon and Galli, 1990). After being activated, MCs rapidly secret granule-stored TNF through degranulation and then release the* de novo* synthesized TNF 24 h later (Zhang B. et al., 2012). TNFα, as a neuro-sensitizing molecule, causes neurogenic inflammation and a lowering of the threshold to stimulation (Wheeler et al., 2014), which may be attributed to activation of cyclooxygenase and the p38 MAP kinase (Zhang et al., 2011). TNFα binds to its receptors and initiates the generation and release of inflammatory mediators produced by immune cells, including MCs (Yang et al., 2018). However, a study of IC pain models that displays an increased number of MCs fails to suggest a role for TNFα in initiating nociception (Rudick et al., 2008).

IL-33 (Martin Jensen et al., 2018) and IL-1beta that secreted from MCs in response to inflammatory molecules, such as lipopolysaccharide and SP, may involve in the processing of local inflammation and hypersensitivity (Coelho et al., 2000; Ebenezer et al., 2018; Taracanova et al., 2018). The neuro-sensitizing effects of some inflammatory cytokines generated and secreted from MCs, such as IL-2, IL-5, IL-6, IL-9, IL-10, IL-11, IL-16, IL-37 and platelet-derived growth factor (Mukai et al., 2018; Conti et al., 2019), need to be validated.

### NGF

NGF is believed to be an important mediator in peripheral hyperalgesia (Pezet and McMahon, 2006). NGF is stored and released by a range of cell types, such as MCs, macrophages, and the sensory and sympathetic neurons (Bandtlow et al., 1987; Liu et al., 2021).

A vitro study reveals that MCs can synthesize, store, and release NGF in response to antigen/IgE stimulation (Leon et al., 1994), while NGF induces human MCs differentiation, maturation, and degranulation (Skaper, 2017). On the one hand, NGF released from MC have profound implications in pain-associated pathobiology, such as osteoarthritis pain (Sousa-Valente et al., 2018) and visceral hypersensitivity (Li et al., 2019). MC-derived NGF may participate in long–lasting peripheral sensitization by governing the enteric synaptic plasticity (Zhang et al., 2018). On the other hand, as MCs express receptors for NGF (Tam et al., 1997), endogenous NGF can elicit the degranulation of MCs, which may be relevant to the early stages of peripheral sensitization and inflammation (Marshall et al., 1990; Groneberg et al., 2005; Sousa-Valente et al., 2018) as well as central sensitization (Kissel et al., 2017).

From the foregoing, it can be concluded that the crosstalk between NGF and MCs may contribute to tissue inflammation and hyperalgesia *via* amplifying each other’s effects. However, the detailed mechanisms of their interaction warrant further research.

## Conclusion

The recent flood of evidence demonstrates the involvement of MCs in painful conditions and suggests a possible mechanism of MCs to pain pathobiology. Noxious stimuli can rapidly activate resident MCs at the injured site, where they release neuro-sensitizing molecules that induce peripheral sensitization, local inflammation, and the recruitment of other immune cells. Meanwhile, MCs interact with mediators that are critical for the maintenance of pain. MCs also modulate nociception centrally *via* enhancing neuronal sensitivity and altering the permeability of the blood-brain barrier (Esposito et al., 2001), allowing the infiltration of additional cells (Figure 2).

The involvement of the immune system in pain appears to be more common than once thought, as common analgesics are often not sufficient to control pain associated with MC activation (Butterfield, 2009; Aich et al., 2015). Systemic MC activation disease (MCAD) is characterized by the accumulation of genetically altered dysfunctional MCs with the abnormal release of these cells’ mediators. Although therapeutic alternatives in MCAD patients with pain are drugs that profoundly stabilize MCs, it remains a challenge considering its adverse effects on human beings (Wirz and Molderings, 2017). Based on the demonstrated efficacy in pain, analgesics that can significantly mitigate MC degranulation, such as morphine (Vincent et al., 2016), *Palmitoylethanolamide* (D’Amico and Impellizzeri, 2020), and ketotifen (Klooker et al., 2010), are promising for treating all those painful conditions in which MC activation is the main cause. Pharmacological targeting of MC proliferation, specific surface antigens, and downstream signaling pathways, in addition to stabilizing MCs, may improve analgesics therapy (Molderings et al., 2016).

Given that MC serves as important source of proinflammatory mediators in sustained nociceptive sensitization, new strategies to manipulate crosstalk between neurons and MC hold considerable promise. However, mechanisms of pain are still emerging, and the molecular mechanisms of MC-mediated pain are worth exploring.

## Author Contributions

WF and LM designed and drafted the manuscript and figures. QL analyzed the data. QL, FH, and HH revised the manuscript. All authors contributed to the article and approved the submitted version.

## Conflict of Interest

The authors declare that the research was conducted in the absence of any commercial or financial relationships that could be construed as a potential conflict of interest.
